# TEAM‐UP: Mixed‐Methods Data for Understanding Traditional and Modern Contraceptive Use Dynamics in Four Sub‐Saharan African Countries

**DOI:** 10.1111/sifp.70046

**Published:** 2026-02-20

**Authors:** Nurudeen Alhassan, Jamaica Corker, Nyovani Janet Madise, Ernestina Coast, Naa Dodua Dodoo, Themba Mzembe, Jacques B.O Emina, Elizabeth Omoluabi, F. Nii‐Amoo Dodoo, Saseendran Pallikadavath, Maame B. Peterson, John A. Mushomi, Funmilola M. OlaOlorun, Eliya Msiyaphazi Zulu

## Abstract

This Data Article describes a novel dataset from the “Re‐Examining Traditional Method Use” (*TEAM‐UP*) project, which systematically collected data on the measurement of and motivations for use of non‐modern (traditional and folkloric) contraceptive methods and/or modern methods, in four sub‐Saharan African countries: the Democratic Republic of Congo, Ghana, Kenya, and Nigeria. TEAM‐UP comprises four datasets (two quantitative and two qualitative), enabling comprehensive analyses of (1) the impact of methodological innovations on reporting of modern and non‐modern method use, and prevalence estimates; (2) motivations for, and user experiences related to traditional and folkloric methods, and (3) contraceptive use dynamics across all methods and method types, including nonuse. Data collection was conducted in four stages: qualitative (Stage 1; 54 focus group discussions and 81 key informant interviews) and quantitative (Stage 2; *n* = 918) pilots, followed by women's surveys (Stage 3; *n* = 13,625) and follow‐up qualitative in‐depth interviews (Stage 4; 469 interviews). The main TEAM‐UP survey data are publicly available, with both the pilot and follow‐up in‐depth qualitative data available upon vetted request.

## INTRODUCTION

Although 92 million women worldwide aged 15–49 years currently use traditional methods of contraception (UNDESA 2022), family planning programmes and research have focused almost exclusively on modern methods. As a result, traditional methods and those who use them are often marginalised in family planning interventions. This is despite the fact that—contrary to the assumption that women and couples use non‐modern methods due to a lack of knowledge about, or poor access to, modern methods—some single‐context studies have shown that many women and couples have a distinct preference for traditional methods even with knowledge of and access to modern contraceptives (Ajayi, Adeniyi and Akpan 2018; Gebreselassie et al. 2017; Namasivayam et al. 2023; Polis et al. 2021; Ram, Shekhar and Chowdhury 2014). Moreover, there is a lack of consensus on how to define “modern” versus “traditional” methods. Some definitions emphasise technology or medical intervention (Hubacher and Trussell 2015), others rely on efficacy (Festin et al. 2016; Moroole et al. 2020), while users often categorise methods by hormones or technology (Rossier, Senderowicz, and Soura 2014). Withdrawal and periodic abstinence, often combined together into a singular category of “traditional” methods, have some efficacy for preventing pregnancy, though less reliable than most modern methods, whereas folkloric practices lack proven efficacy (Fehring 2019). Additionally, some researchers note that labeling methods as “traditional” risks implying inevitable abandonment in favor of modern methods, reflecting historic Western trajectories that may not align with contemporary contexts (Johnson‐Hanks 2002; Rossier and Corker 2017).

A number of recent studies suggest that nationally representative surveys (e.g., Demographic and Health Surveys (DHS), Multiple Indicator Cluster Surveys (MICS), and the Performance Monitoring for Action (PMA)) systematically underestimate prevalence of traditional contraceptive method use (Bertrand, Ross, and Glover 2022; Marston et al. 2017; Polis et al. 2021; Rossier, Senderowicz, and Soura 2014), implying that true levels of traditional method use are higher than currently captured or reported. For instance, in Ouagadougou (Burkina Faso), adding follow‐up questions on the lactational amenorrhea method, rhythm, and withdrawal yielded an estimated prevalence of use of these methods of 26 percent compared to 5 percent reported in DHS for the same area (Rossier, Senderowicz, and Soura 2014). Similarly, probing for fertility‐awareness‐based methods in Ghana estimated the prevalence of rhythm at 13.6 percent compared to the DHS estimate of 4.8 percent (Polis et al. 2021). The apparent underestimation of traditional method use in surveys arises both from how contraceptive use questions are framed and from the practices of reporting only the most effective method. Research shows that using terms like “method” and “using”^1^ in questions about contraceptive use primes respondents to focus on modern methods in their responses rather than to think of their traditional method use, while adding probing questions on non‐modern methods substantially increases their reported use (Polis et al. 2021; Rossier, Senderowicz, and Soura 2014; Staveteig 2017). In addition, the standard approach for estimating overall contraceptive prevalence in surveys is to report only the most effective method when respondents report using more than one method, leading to an underestimation and underreporting of traditional or folkloric method use in cases of concurrent use of both a traditional and modern method^2^ (Gebreselassie et al. 2017; Polis et al. 2021; Rossier, Senderowicz, and Soura 2014; Staveteig 2017). These gaps underscore the need for new data and approaches to improve measurement in order to provide a more comprehensive understanding of non‐modern contraceptive use across multiple settings.

This Data Article describes a new dataset from a cross‐country comparative study on the measurement of and motivations for the use of non‐modern (traditional and folkloric) contraceptive methods in the Democratic Republic of Congo (DRC), Ghana, Kenya and Nigeria. It describes the data from the research study “*Re‐Examining Traditional Method Use” (“TEAM‐UP”*) designed to better measure the true prevalence of traditional method use and to understand women's and men's motivations for using these methods. The TEAM‐UP dataset contains four distinct datasets—two quantitative and two qualitative—enabling comprehensive analyses of (1) the impact of the study's methodological innovations on traditional method use reporting and prevalence; (2) motivations for non‐modern method use and individuals’ understandings of and experiences with traditional and folkloric methods; and (3) contraceptive use dynamics across the full spectrum of approaches to contracepting, including non‐ and never‐use. The study's primary objective was to better understand the full spectrum of contraceptive use for pregnancy prevention, in order to inform family planning programmes and policy. Ancillary exploration of other purposes of contraceptive use, such as sexually transmitted infection (STI) prevention, was included but was not a central focus.

A key aim of TEAM‐UP was to enable the re‐examination and reconsideration of the conventional definitions of contraceptive categories as a modern versus traditional binary, including the interchangeable use of the label “traditional” as an umbrella term for any method considered non‐modern (Gebreselassie et al. 2017; Keogh et al. 2021) or only for withdrawal and periodic abstinence (Bertrand et al. 2022; Rossier and Corker 2017). TEAM‐UP limits the definition of “traditional methods” to withdrawal and periodic abstinence,^3^ because they are the most widely used non‐modern methods and have proven, albeit lower, efficacy than modern methods. This makes “traditional” methods more relevant to family planning programmes than other non‐modern methods categorised as “folkloric,” which have unproven efficacy and are often specific to local contexts, although TEAM‐UP collected data on these methods as well, as they often play a role in contraceptive use dynamics. In this article, we use the umbrella term “non‐modern” to encompass both traditional and folkloric categories. While we have reservations about the terms “modern,” “traditional,” “folkloric,” and “non‐modern,” we use them here because of a lack of viable alternatives that would be widely understood or accepted.

The TEAM‐UP study was a consortium led by the African Institute for Development Policy (AFIDEP) in collaboration with the Regional Institute for Population Studies at the University of Ghana, Akena Associates (Nigeria), College of Medicine at the University of Ibadan (Nigeria), Department of Population and Development Studies at the University of Kinshasa (DRC), Population and Health Research Institute (DRC), and the University of Portsmouth (United Kingdom).

## STUDY DESIGN

### Study Setting

The four countries in the TEAM‐UP study (DRC, Ghana, Kenya, and Nigeria) were selected to generate cross‐country comparative data across settings with diverse patterns of traditional and modern contraceptive use. Within each country, data were collected in two localised study sites: one rural and one urban, in different regions within each country. These sites were intentionally selected from areas (DHS or PMA regions) with contrasting prevalence levels, ensuring that both high and low prevalence of traditional and modern methods were represented (Table 1). Given the limited geographic scope of the study sites, estimates of use cannot be generalized to national or regional levels. Rather, the diversity of study sites provides an opportunity to compare methodological innovations across different countries and contexts (e.g., urban and rural, high and low contraceptive prevalence areas).

**TABLE 1:** Previous measures of contraceptive use and fertility for women 15–49 in the TEAM‐UP study sites, DHS, and PMA

Country	Data and year	tCPR (%)	mCPR (%)	DHS/PMA region	Urban/rural designation	tCPR (%)	mCPR (%)	TFR
DRC	DHS, 2014	11.1	8.1	Kinshasa± (2022)	Urban site	16.2±	25.3±	4.2
				Mai‐Ndombe*	Rural site	15.2	9.5	6.3
Nigeria	DHS, 2018	3.8	10.5	Adamawa	Rural site	5.9	17.9	6.1
				Lagos± (2022)	Urban site	14.3±	26.7±	3.4
Ghana	DHS, 2022	8.0	23.4	Greater Accra	Rural site	6.5	19.0	2.9
				Ashanti	Urban site	6.2	15.5	3.5
Kenya	DHS, 2022	4.6	42.0	Makueni	Rural site	6.2	41.8	3.3
				Mombasa	Urban site	3.2	29.7	2.9

Source: DHS; ±Source PMA

*Rates for Bundundu province, which Mai‐Ndombe was formally part of

*tCPR: Traditional Contraceptive Prevalence Rate

*mCPR: Modern Contraceptive Prevalence Rate

### Data Collection Phases

**FIGURE 1:**
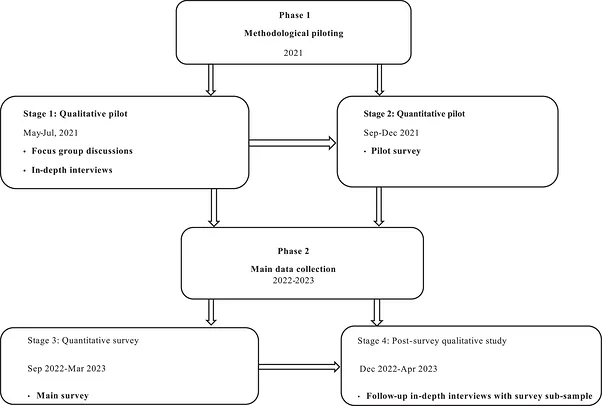
Flow chart of TEAM‐UP study phases

TEAM‐UP data were collected between May 2021 and March 2023. The first phase, including piloting both qualitative (Stage 1) and quantitative (Stage 2) data collection, was followed by the second phase involving women's surveys (Stage 3) and post‐survey qualitative data collection (Stage 4). This produced four distinct datasets: quantitative and qualitative datasets, along with pilot versions of both. Figure 1 summarises the stages of data collection across the two phases.

## PHASE 1: PILOT DATA COLLECTION

### Qualitative Pilot (Stage 1)

This formative phase collected exploratory data on the ways in which “traditional” methods are understood by community members, local leaders (men and women), formal and informal reproductive health providers and users of traditional methods in each study site, as well as how community norms, gendered power relations and structural factors encourage and constrain the use of traditional and folkloric methods. Qualitative methods of in‐depth interviews with key informants (KIIs) and focus group discussions (FGDs) were used to collect the data. The key informants in the in‐depth interviews were female users of traditional and/or folkloric methods, health and family planning providers, including formal health care workers, traditional healers (men and women), birth attendants, and community leaders (men and women). Focus groups were stratified by age, marital status, and sex: older married women (40 years +), older unmarried women (40 years +), younger married women (18‐39 years), younger unmarried women (18‐24 years), older married men (40 years +) and younger unmarried men (18‐24 years). The age split at 40 in the formative phase was intended to capture perspectives from both potential current users (under 40) and potential former users (40 and above) to understand how traditional methods are perceived, and to explore community norms and structural factors shaping their use.

Interview guides for the FGDs and KIIs were developed collectively by the full TEAM‐UP consortium. Separate interview guides were developed and used for the in‐depth interviews with different groups of respondents. The KII guides captured comprehensive data covering the experiences and motivations of traditional or folkloric method users, understanding health system or programmatic factors as well as gendered power relations that support or constrain traditional method use. A single interview guide was used across all FGDs with the aim of exploring community perceptions of traditional contraception, norms around traditional contraceptive use, and ways of engaging community members in TEAM‐UP research, including identifying local names of specific traditional or folkloric methods. All questions in the qualitative pilot were open‐ended, and probes were used as necessary to elicit responses from participants. TEAM‐UP developed a training manual to guide interviewers on how to ask questions and elicit responses using the various interview guides.

Before embarking on data collection, project teams in each of the four countries recruited and trained qualified field staff. Training workshops, ranging from three to four days depending on the country context, introduced field staff to the project and ensured they understood the FGD and KII interview guides and data collection procedures. The training manual provided detailed instructions on how to ask questions across all sections of the guides. As the central focus of the qualitative pilot was to explore how people understood categories of “traditional” and modern contraceptives, interviewers were explicitly trained not to impose definitions of modern, traditional or folkloric methods on participants. Field staff also received training on research ethics, including obtaining informed consent, respecting confidentiality, and participants’ rights to decline participation. As the local languages in these countries are primarily spoken rather than written, the question guides were not translated into local languages for this phase. Instead, the field workers and country research teams worked collectively until a consensus was reached regarding the most accurate and contextually relevant translation of each question. The field workers then practiced asking the questions in the local languages. The training workshops also included practical sessions where field staff conducted mock interviews with team members for feedback and refinement. Only field staff with the appropriate interview skills and competence to administer the question guides according to project design and ethical standards were deployed to conduct the FGDs and KIIs.

Prior to each FGD or KII, written informed consent was obtained from the participant(s). Interviews were conducted in local languages^4^, English, or French, depending on participant(s)’s choice or fluency. A total of 54 FGDs and 81 KIIs were conducted across the four study countries (Table 2). All interviews were audio‐recorded after obtaining participants’ consent, then translated and transcribed into English. Translation and transcription to English were carried out simultaneously for all languages in Ghana, Kenya and Nigeria, with supervisors listening to the recordings and cross‐checking them against the transcribed text for accuracy and quality assurance. In contrast, DRC used a step‐wise transcription and translation: first from local language to French, then from French to English. As a result, the final qualitative pilot data comprise English‐language text transcripts.

**TABLE 2:** Number of qualitative pilot FGDs and KIIs by country

Country	FGDs	KIIs	Total
Ghana	14	22	36
Nigeria	12	20	32
Kenya	16	20	36
DRC	12	19	31
Total	54	81	135

SOURCE: TEAM‐UP Qualitative Pilot Data, May‒July, 2021.

The qualitative pilot data elicited local names and descriptions of traditional methods, along with insights into motivations for using modern and non‐modern contraceptives, concurrent use of modern and “traditional” methods, other sexual practices to prevent pregnancy, and the influence of gender dynamics on contraceptive choices. Although not a primary focus, abortion was raised spontaneously and discussed during conversations about contraceptive use. The results show that participants in both FGDs and KIIs typically associated “traditional” methods with the use of herbs, concoctions, amulets, waist strings, and rings, as well as non‐contraceptive pharmaceuticals. In contrast, “natural methods” were often used to describe withdrawal and periodic abstinence. Findings from the qualitative pilot informed the list of traditional and folkloric methods included in the Stage 2 quantitative pilot questionnaires and helped refine data collection approaches.

### Quantitative Pilot (Stage 2)

The second stage of TEAM‐UP's data collection—the quantitative pilot—sought to address two key methodological questions for the main survey: 1) do modifications to national survey instruments result in different estimates of traditional (non‐modern) method use? and 2) which specific modifications—such as using locally defined method names, prompting and probing, or changing question order—yield different information compared to standard population‐based surveys like the DHS and PMA?

The quantitative pilot tested two versions of a survey instrument (Versions A and B) in the same sites. The comparison sought to: a) assess how different questionnaire content and administration approaches impact estimates of traditional and modern contraceptive use, and b) field‐test additional questions planned for the main survey on contraceptive choice motivations and concurrent use of multiple methods. Version A closely followed standard DHS and PMA formats with only minor modifications: adding two fertility awareness‐based methods (basal body temperature and cervical mucus), asking about contraceptive use in the last 6 months, and asking about use by partner for women with multiple sexual partners. Version B was more experimental: it incorporated all of Version A's modifications but questions began with traditional methods (reversing the DHS sequence) and added probing on knowledge and use of all methods, sexual partners in the past 6 months, motivations for using specific traditional methods, reasons for concurrent use of different methods or method types (e.g., traditional with modern), and method switching and motivations for switching.

Most field staff deployed for the qualitative interviews were retained and trained for implementing the quantitative pilot, with additional staff recruited and trained in some study countries. As in the qualitative pilot, interviewers were trained to administer the pilot questionnaires while adhering to research ethics. For example, the TEAM‐UP phrasing for contraceptive use was: (a) *“Between now and the last 1 month, did you or your partner do anything or try in any way to delay or avoid getting pregnant?”* and, if yes, (b) *“What did you or your partner do in the last 1 month to delay or avoid getting pregnant?”* Interviewers were trained to adhere to the exact wording of the questionnaires and to avoid terms like “method(s)” and “using,” which are common in standard surveys, focusing instead on all ways and things women and couples do to avoid pregnancy, including locally informed concepts or definitions such as “concoction”.^5^ Interviewers were also trained to translate questionnaires from English or French into local languages during interviews.

The sample for the quantitative pilot was drawn randomly from households, using the latest census or survey sampling frames in each study site. Eligible respondents were women aged 15–49 years. One woman per household was selected and randomly assigned to be interviewed using either questionnaire A or B. With a target of 60 women per questionnaire per site, a total of 918 women were interviewed across the four countries for the quantitative pilot (Table 3)

**TABLE 3:** Number of Pilot Interviews Completed per Questionnaire and by Country

Country	Version A	Version B	Total
DRC	121	112	233
Ghana	119	109	228
Kenya	105	107	212
Nigeria	122	123	245
**Total**	**467**	**451**	**918**

SOURCE: TEAM‐UP Quantitative Pilot Data, September‒December 2021.

### Methodological Pilot Data and Findings

The socioeconomic and demographic characteristics of respondents in Versions A and B of the pilot questionnaires were similar across all eight sites (Table 4), providing confidence in the comparability of the data between the two versions.

**TABLE 4:** Socioeconomic and demographic characteristics of TEAM‐UP pilot sample

Sociodemographic characteristics	Version A (*n* = 467)	Version B (*n* = 451)
Age (mean)	29.1 years	29.5 years
Children Ever Born (mean)	3.3	3.3
Marital status (%)		
Never married	34.7	32.6
Currently married	58.2	57.9
Formerly married	7.1	9.6
Educational attainment (%)		
No education	3.2	4.4
Primary	27.2	25.7
Post‐primary/Vocational	19.5	17.9
Secondary	37.4	40.0
Post‐secondary	12.7	12.0
Residence (%)		
Rural	41.5	39.7
Urban	58.4	60.4

SOURCE: TEAM‐UP Quantitative Pilot Data, September‒December 2021.

Analysis of the quantitative pilot data found no statistically significant difference in the percentage of women using traditional and modern methods between the two pilot questionnaires (Table 5), suggesting that the order of probing questions (starting with either traditional or modern methods) did not affect reporting. However, version B showed a slightly higher percentage of women reporting use of traditional and modern methods.

**TABLE 5:** Percent of women using traditional and modern methods by questionnaire version

Contraceptive use	Version A (*n* = 467)	Version B (*n* = 451)	Overall (*n* = 918)	*p*‐value^a^
	*n* (%)	*n* (%)	*n* (%)	
Any modern method	130 (27.8)	137 (30.4)	267 (29.1)	0.489
Traditional method	24 (5.1)	28 (6.2)	52 (5.7)	
Not using any method	313 (67.0)	286 (63.4)	599 (65.3)	

SOURCE: TEAM‐UP Quantitative Pilot Data, September‒December 2021. ^a^Pearson's chi‐squared test.

## PHASE 2: MAIN SURVEY AND QUALITATIVE DATA COLLECTION

### Quantitative Women's Survey (Stage 3)

The main quantitative women's survey adopted version B of the pilot questionnaire because it provided more in‐depth data on contraceptive use dynamics and motivations while maintaining aspects that facilitate comparisons with different survey approaches (e.g., DHS). As the principal rationale of the TEAM‐UP design was to examine how modifications to standard survey instruments influence estimates of traditional and modern contraceptive prevalence, the Version B questionnaire was determined to be best placed to test these methodological innovations. In addition to estimating prevalence, this version of the questionnaire also captured more detailed data on the motivations behind contraceptive choices, particularly around the use of traditional and folkloric methods, and allowed for interrogation of reasons for concurrent use of non‐modern and modern methods. It also captured profiles of various user types—consistent traditional method users, concurrent users, and switchers across and between method types—and included experimental questions on pregnancy risk perceptions and intentions. As a result, Version B questionnaire was selected for the main survey as it would better generate new and improved data to inform the improved measurement of the full range of contraceptive use that was the central aim of TEAM‐UP.

The key modifications incorporated in the main survey questionnaire were as follows:

- *Follow‐up questions on contraceptive method use were asked of all respondents*: In contrast to surveys such as DHS and PMA, TEAM‐UP asked follow‐up questions on contraceptive use to all women—including those who did not initially or spontaneously report using any of the listed contraceptive methods. The aim of this modification in TEAM‐UP was to reduce underreporting of non‐modern methods, which can occur due to recall bias or respondents interpreting the initial question on contraceptive use as pertaining only to modern methods.
- *Probing questions on use*: Unlike DHS and PMA, which probe only for method knowledge, the TEAM‐UP survey asked probing questions about use for all methods. This allowed for the recording of all methods cited by respondents and calculating the prevalence of concurrent or sequential use over the past month and 6 months.
- *Differentiating methods reported spontaneously versus after probing*: TEAM‐UP data distinguished between methods that were spontaneously mentioned and those reported only after probing. This allows for analysis of which methods are most likely to be underreported in surveys that do not include comprehensive probing.
- *Data on current, recent, and ever‐use*: TEAM‐UP provides data on all methods, including multiple methods, used by respondents during two time periods: *current use* (in the past 4 weeks) and *recent use* (within the past 6 months), as well as *ever use* (at any time in the past)^6^.
- *Experimental questions on fertility desires*: TEAM‐UP included experimental questions^7^ ^8^ on the importance of delaying pregnancy, perceived pregnancy risk, and the intention to use any contraceptive in the next 12 months among nonusers. These questions can be used to analyse contraceptive use, nonuse, and decision‐making for both modern and traditional methods.
- *Data on other sexual practices for pregnancy avoidance and contraceptive use for other purposes*: The main survey also asked about non‐penile‐vaginal practices such as masturbation, oral sex, anal sex, intercrural sex, and use of sex toys or aids that women or couples might use to delay or prevent pregnancy, as well as questions on contraceptive use for STI protection and to manage menstruation in order to capture the full range of motivations for contraceptive use including and beyond pregnancy prevention.

Given the above modifications to the TEAM‐UP study design, workshops were organised to retrain field staff to understand the questionnaire and approaches for interviewing respondents. The workshops provided clarity on field procedures and ensured consistency in the training. In addition to the consortium‐wide workshops, each country team organised its own training workshop(s) in preparation for data collection. The draft questionnaire was reviewed by members of the TEAM‐UP International Scientific Advisory Group and pretested before it was finalised and deployed for data collection.

#### Sampling for the Main Women's Survey

The target sample size for the main TEAM‐UP survey was 13,701 women aged 15‒49 years from all the study countries. The sample was drawn using a multi‐stage sampling strategy. A gridded population sampling technique was employed to generate sample frames, given the lack of recent survey/census sampling frames in some of the study countries—particularly DRC. The study sites were divided into clusters based on their gridded population data, using ArcGIS 10.6 parameters and the Gridsample algorithm in the R statistical package (Thomson et al. 2020). The first stage of sampling involved a random selection of the clusters generated through the gridded sampling technique, without stratification. To achieve the desired sample size, we randomly selected a total of 271 clusters across the four countries, with a variable take of between 30 and 60 households per cluster (depending on the type (urban/rural) and specificities (e.g., linguistic complexity, density) of the site). The total number of clusters selected in the first stage of sampling consisted of 61 clusters in DRC, 47 in Ghana, 60 in Nigeria, and 103 in Kenya.

Following the random selection of clusters, a complete listing of households in each cluster was conducted to form the sample frame for the household selection. The list of households in each cluster was generated using a listing form designed by TEAM‐UP. The number of households randomly selected in each cluster for the household interviews ranged between 30 and 60, depending on site characteristics and complexities. The final stage involved the identification of eligible respondents in the selected households. A complete list of household members was generated from interviewing household heads or an adult household member capable of responding to the household questionnaire. The household questionnaire included information about the age, sex, and residence status (usual resident or visitor) of each household member. All eligible respondents (women aged 15–49 years who were usual residents of the household) were interviewed using the women's questionnaire. Even though similar field protocols were implemented across countries, the differences in the response rate were likely due to contextual differences in study sites. The final survey data contains information from 13,625 eligible women from 13,263 households across the four countries (Table 6).

**TABLE 6:** Sample distribution of households and women in the main TEAM‐UP survey

Country	Site	Total HH selected and successfully interviewed	Total number of women (15‐49 years) in the selected households	Number of women interviewed	Women's response rate (%)^a^
Nigeria	Lagos	2,340	2,032	1,900	93.5
	Adamawa	1,200	1,525	1,505	98.7
		**3,540**	**3,557**	**3,405**	**95.7**
DRC	Kinshasa	1,941	2,696	2,351	87.2
	Mai‐Ndombe	1,591	1,923	1,664	86.5
		**3,532**	**4,619**	**4,015**	**86.9**
Ghana	Accra	1,208	1,061	967	91.1
	Ashanti	1,922	1,876	1,620	86.4
		**3,130**	**2,937**	**2,587**	**88.1**
Kenya	Makueni	1,160	1,531	1,325	86.5
	Mombasa	1,901	2,719	2,293	84.3
		**3,061**	**4,250**	**3,618**	**85.1**
Total		13,263	15,363	13,625	88.7

SOURCE: TEAM‐UP Main Survey Data, September 2022‒March 2023.

^a^(Number of women interviewed/total number of women in selected households) *100%.

**TABLE 7:** Socioeconomic and demographic characteristics of all women aged 15–49 years in TEAM‐UP women's survey sample (weighted)

Background characteristics	DRC	Ghana	Kenya	Nigeria
	(n = 4,015)	(*n* = 2,587)	(*n* = 3,618)	(*n* = 3,405)
	*n* (%)	*n* (%)	*n* (%)	*n* (%)
Age (years)				
15–19	938 (21.0)	522 (20.2)	390 (9.6)	610 (16.2)
20–24	919 (22.2)	480 (18.2)	783 (22.6)	575 (14.9)
25–29	677 (18.0)	377 (15.1)	769 (22.2)	535 (15.6)
30–34	513 (13.0)	390 (15.7)	546 (15.3)	483 (14.0)
35–39	475 (12.7)	339 (13.3)	511 (14.1)	500 (16.8)
40–45	287 (7.6)	275 (9.9)	357 (9.2)	415 (12.9)
45–49	206 (5.5)	204 (7.6)	262 (7.0)	287 (9.6)

Place of residence				
Rural	1,744 (23.4)	1,902 (75.1)	1,086 (21.8)	1,288 (21.5)
Urban	2,271 (76.6)	685 (24.9)	2,532 (78.2)	2,117 (78.5)

Education				
No education	266 (4.0)	394 (14.2)	137 (4.6)	488 (6.95)
Primary	556 (10.6)	664 (25.5)	1,556 (44.2)	480 (10.9)
Secondary	2,715 (69.5)	1,391 (54.7)	1,384 (37.5)	1,709 (55.6)
Higher	478 (15.9)	138 (5.6)	541 (13.8)	728 (26.6)

Religion				
Catholic	754 (13.0)	94 (3.2)	639 (16.5)	143 (4.4)
Protestant	623 (10.8)	380 (13.3)	1,037 (24.6)	305 (8.6)
Charismatic	514 (13.7)	1,118 (45.9)	812 (23.2)	553 (23.4)
Other Christian	1,585 (49.4)	607 (24.6)	255 (8.7)	866 (26.3)
Islam	28 (0.4)	302 (9.7)	847 (26.1)	1,520 (36.8)
None/Other	511 (12.6)	86 (3.3)	28 (0.8)	18 (0.5)

Marital status				
Never in union	1,434 (40.6)	934 (37.2)	909 (23.5)	1,055 (31.3)
Currently married	1,769 (41.0)	1,078 (40.5)	2,154 (59.9)	2,009 (58.4)
Living together	404 (12.3)	402 (15.7)	152 (4.9)	84 (3.3)
Divorced/Widow	254 (6.1)	164 (6.6)	398 (11.7)	246 (7.0)

SOURCE: TEAM‐UP Main Survey Data, September 2022‒March 2023.

### Key Findings From the Main TEAM‐UP Women's Survey Data

The data comprises information on the following: (a) socioeconomic and demographic characteristics of the survey respondents, (b) sexual activity and use of modern and non‐modern contraceptives, (c) pregnancy intention and knowledge of ovulatory cycle, and (d) fertility desires and future intentions to use contraceptives (see Table 7). Data on some socioeconomic and demographic characteristics of respondents from TEAM‐UP are comparable to data from other surveys, but with some slight differences.^9^^,^ 10 ^,^ 11

The results of the methodological innovations tested revealed that probing and follow‐up questions increased reporting of current use of both traditional and modern methods across sites (Table 8). Across all sites, probing resulted in higher reported use of modern and traditional methods and a corresponding decrease in the share of women reporting nonuse, though there was some notable variation—although across all sites, the greatest proportional increase was for traditional methods. Adamawa (Nigeria) saw the greatest increases, with probing doubling the percentage of women using modern methods as well as the percentage using traditional methods (although both were still among the lowest across all sites). Differences across sites in post‐probing changes in method reporting probably reflect local norms surrounding sexuality, privacy, and contraceptive communication that influence how probing elicits reports of use—or differences in interviewer probing. While some variation in interviewer probing cannot be ruled out, the standardised training, implementation, and supervision for the data collection were designed to ensure consistency across sites. Taken together, the results (Table 8) suggest that exhaustive probing can increase reporting of method use without introducing new questions into current survey designs.

**TABLE 8:** Percentage of women who report using any traditional, or any modern, contraception before and after probing, by study site^a^

			Prevalence before probing			Prevalence after probing		
		Number of women	*n* (%)			*n* (%)		
Country	Site	(*n*)	Any modern	Traditional	Not using	Any modern	Traditional	Not using
DRC	Kinshasa	2,351	407 (17.0)	147 (6.1)	1,797 (76.9)	649 (27.7)	187 (7.9)	1,515 (64.4)
	Mai‐Ndombe	1,664	80 (5.1)	118 (7.1)	1,466 (87.8)	119 (7.7)	174 (10.6)	1,371 (81.7)
Ghana	Ashanti	1,620	305 (19.1)	30 (1.9)	1,285 (79.1)	341 (21.5)	52 (3.2)	1,227 (75.3)
	Accra	967	162 (16.7)	44 (5.1)	761 (78.2)	207 (21.4)	57 (5.8)	703 (72.8)
Kenya	Makueni	1,325	646 (48.6)	73 (5.2)	606 (46.2)	701 (52.5)	88 (6.5)	536 (41.0)
	Mombasa	2,293	891 (40.5)	59 (2.5)	1,343 (57.0)	922 (41.9)	73 (3.1)	1,298 (55.0)
Nigeria	Lagos	1,900	321 (16.7)	167 (10.2)	1,412 (73.1)	368 (19.3)	196 (11.6)	1,336 (69.1)
	Adamawa	1,505	96 (6.2)	19 (1.5)	1,390 (92.3)	190 (12.5)	36 (2.7)	1,279 (84.8)

SOURCE: TEAM‐UP Main Survey Data, September 2022‒March 2023. ^a^Totals are over 100, as respondents who reported the use of one or more traditional as well as one or more modern methods were counted in both categories (i.e., counted as both modern method and traditional method users).

### Post‐Survey Qualitative Study (Stage 4)

The final stage of data collection involved qualitative in‐depth interviews (IDIs) using a semistructured guide to gather more in‐depth information on influences, reasons, and motivations for use of non‐modern methods (withdrawal, periodic abstinence, herbs, and concoctions), including concurrent and/or sequential use. Interviews were conducted with a subsample of women survey respondents (Stage 3) and with male partners of the respondents.^12^ Data collection took place from December 2022 to April 2023. Interviewers in each country were trained on the interview guides, research ethics, and qualitative interviewing techniques.

The post‐survey qualitative study aimed to interview 40 women and 15 men in each site, for a target of 80 women and 30 men in each country and a total goal of 320 interviews with women and 120 with men across all sites. For women, four predefined profiles were used to select respondents from the survey for the qualitative study (Table 9).

**TABLE 9:** Predefined profiles for selecting non‐modern method users for the qualitative interviews

Predefined profiles of non‐modern (traditional) method users	Description of profiles
1. Consistent users	Women using non‐modern methods consistently in the 6 months preceding the survey. This could include using periodic abstinence on “unsafe” days and withdrawal on “safe” days.
2. Concurrent users	Women who reported using both non‐modern and modern method(s) within the same 1‐month period.
3. Switchers	Women who switched from non‐modern to modern method(s) or vice‐versa at some point during the 6 months preceding the survey.
4. Stoppers	Women who previously (2 to 6 months preceding the survey) used non‐modern methods, but had used no method in the past 1 month.

In each site, 10 women from each of the four profiles above were selected for in‐depth interviews. In addition, male partners of some of the women interviewed in the qualitative study were also interviewed. In total, 337 women and 132 male partners were interviewed in the post‐survey qualitative study (Table 10).

**TABLE 10:** Number of IDIs completed in each country, by gender

Gender	Ghana	Kenya	Nigeria	DRC	Total
Women	110	77	77	73	337
Men	44	34	34	20	132
Total	154	111	111	93	469

SOURCE: TEAM‐UP Post‐Survey Qualitative Data, December 2022‒April 2023.

The qualitative in‐depth interviews were audio‐recorded with the consent of the respondents. The audio records were translated and transcribed using the same approach in the qualitative pilot, as previously described.

### TEAM‐UP Data Value and Usage Opportunities

TEAM‐UP provides comprehensive quantitative and qualitative data on the full range of contraceptive methods that can address key research and policy questions on contraceptive use and inform improvements to measuring contraceptive prevalence through analyses by researchers, policymakers, and programmers. Table 11 summarizes the TEAM‐UP datasets and their key analyses opportunities.

**TABLE 11:** Summary of TEAM‐UP datasets

Data collection stages	Data type	Analysis opportunities
1. Qualitative pilot	FGD and KII transcripts on how traditional and folkloric contraceptive methods are understood, defined, labelled, used and perceived across eight sites.	Analysis of contraceptive definitions and classifications. Social norms and expectations around modern and traditional contraceptive use. Discussions around abortion in the context of contraceptive use.
2. Quantitative pilot	Quantitative data (raw survey data in STATA format) comparing questionnaire content and administration effects on reporting non‐modern and modern contraceptive use.	Effect of probing and probing sequence on contraceptive estimates.
3. Quantitative Survey	Quantitative data (anonymized survey data in STATA format) on women's contraceptive use motivations, intentions, and behaviors, and multiple approaches to measuring fertility desires.	Contraceptive prevalence estimates and comparisons (for one or 6 months preceding the survey). Analysis of different measures of concurrent contraceptive use. Comparisons of different questions on fertility preference and their relation to contraceptive use.
4. Post‐survey qualitative study	Text‐based transcripts (in‐depth interviews) from a subsample of women and their partners, providing detailed information on specific patterns of contraceptive use, motivations and patterns	Personal perspectives and experiences of contraceptive use (method type, consistency, concurrency, switching, stopping) linked to quantitative data. Motivations and explanations for non‐/contraceptive choices. Motivations and reasons for use of traditional, modern and folkloric methods. Exploration of pre‐and post‐coital contraception and abortion. Influence of decision‐making dynamics on contraceptive choice and use.

The release of this comprehensive data on contraceptive use dynamics and motivations across the full spectrum of methods is particularly timely given the suspension of the DHS and current dialogues around the future of survey data. As the FP field must now explore new approaches to collecting data on contraceptive use, family planning, and sexual and reproductive rights, the TEAM‐UP data can inform methodological improvements to better capture the full range of methods and approaches women and men employ to prevent pregnancy and improve current estimation approaches to measuring contraceptive use.

## LIMITATIONS

The TEAM‐UP dataset is not representative beyond the survey sites and should not be used to describe populations at the country or regional level. While comparisons with other data—that is, when DHS clusters overlap with TEAM‐UP sites—are possible, the study's cross‐country approach was designed to test data collection methods and innovative survey questions, rather than to provide population‐level data. Additionally, compared with our results, other studies in the region have found greater increases in reported traditional method use following probing (e.g., Polis et al. 2021; Rossier, Senderowicz, and Soura 2014), suggesting that probing may, in some cases, encourage over‐reporting through social desirability effects. Although our findings confirm that probing increases reported use—especially for non‐modern methods—the inter‐site differences within the TEAM‐UP data and discrepancies with previous studies point to the need for further methodological refinement and validation across diverse settings.

## AUTHOR CONTRIBUTIONS

**NA**: Conceptualization, methodology, original drafting and project administration. **JC**: Conceptualization, methodology, and writing‒reviewing and editing. **NJM**: Conceptualization, writing‒reviewing and editing, and project principal investigator. **EC**: Writing‒reviewing and editing, and supervision. **NDD**: Writing‒reviewing and editing, and project administration. **TM**: Quantitative data curation and writing‒reviewing and editing. **JE**: Writing‒reviewing and editing, and project administration. **EO**: Writing‒reviewing and editing, supervision, data curation and project administration. **FND**: Writing‒reviewing and editing, and supervision. **SP**: Writing‒reviewing and editing, and supervision. **MBP**: Writing‒reviewing and editing. **JAM**: Writing‒reviewing and editing. **FO**: Writing‒reviewing and editing, and supervision. **EMZ**: Writing‒reviewing and editing, and funding acquisition.

## FUNDING INFORMATION

The TEAM‐UP project was supported by the Bill & Melinda Gates foundation under grant INV‐006353.

## CONFLICT OF INTEREST STATEMENT

The authors declare that the research was conducted in the absence of any commercial or financial relationships that could be construed as a potential conflict of interest.

## ETHICAL STATEMENT

Ethical approval for the TEAM‐UP study was granted by review boards in the four countries. In Kenya, by AMREF Ethics and Scientific Review Committee (Ref #: AMREF─ESRC P945/2021) and the National Commission for Science, Technology and Innovation (License #: NACOSTI/P/21/10530); in Nigeria by the National Health Research Ethics Committee of Nigeria (Approval #: NHREC/01/01/2007‐15/03/2021); in DRC by the Comité National d'Éthique de la Santé (National Committee of Health Ethics, Ministry of Health) under No. 259/CNES/BN/PMMF/2021 du1er /06/2021; and in Ghana, by the University of Ghana's Ethics Committee for the Humanities (approval #: ECH 131.20‐21).

## Data Availability

The main TEAM‐UP survey data [anonymized survey data stored in STATA format], the women's questionnaire and the data use dictionary are publicly available at https://afidep.org/publication/release‐of‐team‐up‐data/. Redacted qualitative data [text‐based transcripts] are available upon written request to the Executive Director of AFIDEP. The pilot questionnaire, qualitative data collection tools, and training manuals developed by the TEAM‐UP consortium are publicly available via OSF at https://osf.io/cs5md/.

## Footnotes

1 The main questions on current contraceptive use in standard surveys such as DHS and PMA are worded as follows: (a) “*Are you or your partner currently doing something or using any method to delay or avoid getting pregnant*” and, if yes, (b) “*Which method are you using*?”

2 For example, Polis et al. (2021) found that approximately 23 percent of rhythm users in Ghana would be classified as (only) condom or emergency contraceptive (EC) users under the standard estimation approach, even if they use rhythm as their primary method with occasional use of condom or emergency contraception (EC).

3 This definition also bypasses the debate about categorization for lactational amenorrhea method and the standard days method, which TEAM‐UP classifies as modern due to their low usage, minimal impact on prevalence, and the study's focus on methods generally excluded from programs.

4 Interviews were conducted in the following local languages: DRC (Lingala), Nigeria (Yoruba and Hausa), Ghana (Ga, Dangbe, Ewe, and Ashanti Twi), and Kenya (Swahili and Kamba).

5 The probe for concoctions is worded as follows: “A combination of various ingredients, including herbs, spices, pharmaceutical products, condiments or powdery substances dissolved, milled or mixed up together that a person may drink, insert into the body or apply externally on body parts to prevent pregnancy.”

6 All major FP surveys collect data on current use, though the questions do not define “current,” and while the DHS calendar can be used to calculate contraceptive use in the past 6 months, it does so only for one method at a time and does not capture concurrent use. DHS previously collected data on ever use but discontinued this for most surveys around 2008. PMA currently collects data on ever use

7 If you were to get pregnant today, how would you feel about it?

8 Even though you are currently NOT doing anything to delay or avoid getting pregnant, would you like to be doing something now or using any strategy to delay or avoid getting pregnant?

9
*Education*: DRC: 85 percent have secondary or higher education, which is close to the 88.9 percent in the last DRC DHS. Nigeria: 82 percent in our sample have at least some secondary education, slightly lower than the 89.9 percent in the 2021 Malaria Indicator Survey. Kenya: 51 percent of the pooled sample have at least some secondary education, compared to 58 percent nationally in the 2022 DHS. The percentage with no education (4.6 percent) is close to the national figure (5.5 percent). Ghana: 60 percent of women across our two sites have secondary or higher education, compared to 70 percent nationally in the 2022 DHS.

10
*Marital status*: DRC: 41 percent of the TEAM‐UP sample are currently married, slightly lower than the 46.5 percent reported in the 2014 DHS; 12.3 percent are living with a partner compared to 17.7 percent in the 2022 DHS. 40.6 percent have never been married, higher than the 26 percent in DHS. Ghana: 40.5 percent are currently married, similar to the 40 percent in the 2022 DHS. 15.7 percent are living with a partner (slightly higher than the 14.6 percent in DHS). 37.2 percent have never been married, similar to the 35.1 percent in DHS. Kenya: 59.9 percent are currently married, higher than the 48.1 percent in the 2022 DHS. 23.5 percent have never been married, lower than the 32.5 percent in DHS.

11
*Residence*: the TEAM‐UP sample in DRC, Nigeria, and Kenya was disproportionately urban because the sites were deliberately selected to include large cities in these three countries. The Ghana sample, on the other hand, had high rural residents because rural Accra was deliberately selected due to the high traditional method prevalence and patrilineal descent which also had implication on reproductive behavior including contraceptive use.

12 Women interviewed in the survey were asked if they were willing to participate in a follow‐up interview for themselves and for their partners. The male and female qualitative interviews were designed to provide insights from both men and women in the same area, but not to link responses between partners.

## References

[sifp70046-bib-0001] Ajayi, Anthony I. , Oladele Vincent Adeniyi , and Wilson Akpan . 2018. “Use of Traditional and Modern Contraceptives among Childbearing Women: Findings from a Mixed Methods Study in Two Southwestern Nigerian States.” BMC Public Health 18 (604): 1–9. 10.1186/s12889-018-5522-6 PMC5941455 29739372

[sifp70046-bib-0002] Bertrand, Jane T. , John Ross , and Annie L. Glover . 2022. “Declining Yet Persistent Use of Traditional Contraceptive Methods in Low‐ and Middle‐Income Countries.” Journal of Biosocial Science 54 (5): 742–759. 10.1017/S0021932021000341 34269170 PMC11753471

[sifp70046-bib-0003] Fehring, Richard J. 2019. “Efficacy of Natural Family Planning Methods.” Ethics & Medics 44 (12): 1‒4. https://www.ncbcenter.org/ethics‐and‐medics

[sifp70046-bib-0004] Festin, Mario Philip R. , James Kiarie , Julie Solo , Jeffrey Spieler , Shawn Malarcher , Paul F.A. Van Look , and Marleen Temmerman . 2016. “Moving Towards the Goals of FP2020 — Classifying Contraceptives.” Contraception, 94 (4), 289–294. 10.1016/j.contraception.2016.05.015 27287693 PMC5032916

[sifp70046-bib-0005] Gebreselassie, Tesfayi , Kristin Bietsch , Sarah Staveteig , and Thomas Pullum . 2017. “Trends, Determinants, and Dynamics of Traditional Contraceptive Method Use.” DHS Analytical Studies 63: 1‒75. ICF Rockville, Maryland, USA.

[sifp70046-bib-0006] Hubacher, David , and James Trussell . 2015. “A Definition of Modern Contraceptive Methods.” Contraception 92 (5): 420–421. 10.1016/j.contraception.2015.08.008 26276245

[sifp70046-bib-0007] Johnson‐Hanks, Jennifer. 2002. “On the Modernity of Traditional Contraception: Time and the Social Context of Fertility.” Population and Development Review 28 (2): 229–249.

[sifp70046-bib-0008] Keogh, Sarah C. , Easmon Otupiri , Philicia W. Castillo , Doris W. Chiu , Chelsea B. Polis , Emmanuel K. Nakua , and Suzanne O. Bell . 2021. “Hormonal Contraceptive Use in Ghana: The Role of Method Attributes and Side Effects in Method Choice and Continuation.” Contraception 104 (3): 235–245. 10.1016/j.contraception.2021.05.004 33992609

[sifp70046-bib-0009] Marston, Cicely , Alicia Renedo , Gertrude Nsorma Nyaaba , Kazuyo Machiyama , Placide Tapsoba , and John Cleland . 2017. “Improving the Measurement of Fertility Regulation Practices: Findings from Qualitative Research in Ghana.” International Perspectives on Sexual and Reproductive Health 43 (3): 111–119. 10.1363/43e4517 29553472

[sifp70046-bib-0010] Moroole, Molelekwa A. , Simeon A. Materechera , Wilfred Otang‐Mbeng , and Adeyemi O. Aremu . 2020. “African indigenous contraception: A review”. African Journal of Reproductive Health 24 (4): 173‒184. 10.29063/ajrh2020/v24i4.18 34077082

[sifp70046-bib-0011] Namasivayam, Vasanthakumar , Bidyadhar Dehury , Ravi Prakash , Marissa Becker , Preeti Anand , Ashish Mishra , Shreya Singhal , Shivalingappa Halli , James Blanchard , Dean Spears , and Shajy Isac . 2023. “Understanding the Rise in Traditional Contraceptive Methods Use in Uttar Pradesh, India.” Reproductive Health 20 (8): 10.1186/s12978-022-01547-y PMC9817250 36609308

[sifp70046-bib-0012] Polis, Chelsea B. , Easmon Otupiri , Suzanne O. Bell , and Roderick Larsen‐Reindorf . 2021. “Use of Fertility Awareness‐Based Methods for Pregnancy Prevention Among Ghanaian Women: A Nationally Representative Cross‐Sectional Survey.” Global Health: Science and Practice 9 (2): 318–331. 34234024 10.9745/GHSP-D-20-00601 PMC8324203

[sifp70046-bib-0013] Ram, Faujdar , Chander Shekhar , and Biswabandita Chowdhury . 2014. “Use of Traditional Contraceptive Methods in India & Its Socio‐Demographic Determinants.” Indian Journal of Medical Research 140 (Supplement): 17–28. PMC4345748 25673538

[sifp70046-bib-0014] Rossier, Clémentine , and Jamaica Corker . 2017. “Contemporary Use of Traditional Contraception in Sub‐Saharan Africa.” Population and Development Review 43 Suppl (1): 192–215. 10.1111/padr.12008 29307923 PMC5749414

[sifp70046-bib-0015] Rossier, Clémentine , Leigh Senderowicz , and Abdramane Soura . 2014. “Do Natural Methods Count? Underreporting of Natural Contraception in Urban Burkina Faso.” Studies in Family Planning 45 (2): 171–182. 10.1111/j.1728-4465.2014.00383.x 24931074

[sifp70046-bib-0016] Staveteig, Sarah. 2017. “Fear, Opposition, Ambivalence, and Omission: Results from a Follow‐up Study on Unmet Need for Family Planning in Ghana.” PLoS ONE 12 (7): e0182076. 10.1371/journal.pone.0182076 PMC5536298 28759624

[sifp70046-bib-0017] Thomson, Dana R. , Dale A. Rhoda , Andrew J. Tatem , and Marcia C. Castro . 2020. “Gridded Population Survey Sampling: A Systematic Scoping Review of the Field and Strategic Research Agenda.” International Journal of Health Geographics 19 (34): 1–16. 10.1186/s12942-020-00230-4 32907588 PMC7488014

[sifp70046-bib-0018] UNDESA . 2022. “World Family Planning 2022: Meeting the Changing Needs for Family Planning: Contraceptive Use by Age and Method”. *UN DESA/POP/2022/TR/NO. 4*. New York.

